# Protection by *Vitis vinifera* L. Against Cisplatin-Induced Testicular Injury: Oxidative Stress, Inflammation, and Ferroptosis

**DOI:** 10.3390/ph19010178

**Published:** 2026-01-20

**Authors:** Salman A. A. Mohammed, Hebatallah M. Saad, Kariman A. Esmail, Duaa Eliwa, Aya H. Rohiem, Amal A. Awad, Samar A. El-Adawy, Shimaa S. Amer, Ehab Y. Abdelhiee

**Affiliations:** 1 Department of Pharmacology and Toxicology, College of Pharmacy, Qassim University, Buraydah 51452, Saudi Arabia; 2 Department of Pathology, Faculty of Veterinary Medicine, Matrouh University, Marsa Matrouh 51744, Egypt; heba.magdy@mau.edu.eg; 3 Department of Physiology, Faculty of Veterinary Medicine, Alexandria University, Alexandria 21527, Egypt; kariman.esmail@alexu.edu.eg (K.A.E.); ayahosnyvet@alexu.edu.eg (A.H.R.); 4 Department of Pharmacognosy, Faculty of Pharmacy, Tanta University, Tanta 31527, Egypt; doaa.aleiwa@pharm.tanta.edu.eg; 5 Department of Veterinary Pharmacology, Faculty of Veterinary Medicine, Alexandria University, Alexandria 21527, Egypt; amalali@alexu.edu.eg; 6 Department of Biochemistry, Faculty of Pharmacy, Tanta University, Tanta 31527, Egypt; samar.eladawy@pharm.tanta.edu.eg; 7 Department of Theriogenology, Faculty of Veterinary Medicine, Matrouh University, Marsa Matrouh 51744, Egypt; shimaa.samy@mau.edu.eg; 8 Department of Forensic Medicine and Toxicology, Faculty of Veterinary Medicine, Matrouh University, Marsa Matrouh 51744, Egypt; ehabyahya76@mau.edu.eg

**Keywords:** grape seed extract, cisplatin, rats, testis, inflammation, antioxidant

## Abstract

**Background/Objectives**: Testicular toxicity is one of the most important chemotherapeutic adverse effects of Cisplatin (Cisp), which restricts its use and effectiveness. This study investigated the preventive effects of *Vitis vinifera* L. extract on Cisp-induced testicular injury in rats. **Methods**: Forty adult albino male rats were allocated into four groups: control, *Vitis vinifera* L. extract, Cisp, and co-treated (*Vitis vinifera* L. extract + Cisp). Sperm motility and count, serum reproductive hormones, oxidative/antioxidant biomarkers, pro-inflammatory cytokines, ferroptosis biomarkers, and gene expression profiles were evaluated. **Results**: Cisp administration markedly impaired reproductive performance, as evidenced by significant declines in serum FSH, LH, testosterone, and sperm motility and count. Cisp also induced oxidative stress by elevating MDA, GSSG, GPx, and 8-OHdG, while reducing SOD, Catalase, NRF2, and Ho-1 along with total and reduced GSH levels. Moreover, it triggered strong inflammatory responses and ferroptosis activation, with notable up-regulation of NFκB, TNF-α, IL-1β, ferritin, and cathepsin. Gene expression analysis revealed down-regulation of ARNTL, PI3K, and miR-125b and up-regulation of ASCL4, GSK3B, and COX2 following Cisp exposure. Conversely, co-treatment with *Vitis vinifera* L. extract significantly ameliorated these alterations, restoring sperm quality, hormone balance, antioxidant defenses, and modulating inflammatory, ferroptosis, and genetic responses toward normalcy in addition to restoring testicular and epididymal histoarchitecture without any significant effect in NRF2 and ARNTL expression. Additionally, co-treated groups with *Vitis vinifera* L. extract showed a significant decline in NF-kB p65 and increased PCNA testicular immunoreactivity with a substantial down-regulation in NF-kB p65 and PCNA epididymal immunoreactivity. *Vitis vinifera* L. extract alone did not affect any studied parameters as compared to the control group. **Conclusions**: These findings suggested that *Vitis vinifera* L. extract has a significant protective effect against Cisp-related testicular injury through antioxidative, anti-inflammatory, and anti-ferroptotic mechanisms.

## 1. Introduction

Infertility remains a common problem that calls for continued investigation into effective treatment options. Certain chemotherapy drugs, like Cisplatin (Cisp), have been shown to cause temporary infertility depending on how they are administered [1,2]. Cisp is a commonly used antineoplastic medication that is effective against a variety of solid tumors. However, its therapeutic benefits are often limited by significant toxicity. Cisp functions as an alkylating-like agent that interferes with DNA, producing strand damage and initiating the production of reactive oxygen species (ROS). These effects extend beyond tumor cells, impacting both the endocrine and exocrine systems. In males, Cisp exposure has been linked to impaired androgen production, disruption of gonadal function, and altered spermatogenesis. Clinically, this is reflected in reduced sperm motility, abnormal sperm morphology, and chromosomal defects in spermatozoa [3,4]. The oxidative stress plays a central role, with redox imbalance disrupting cellular metabolism and increasing lipid peroxidation, ultimately damaging germ cells [5]. Among the many side effects of Cisp therapy, testicular toxicity remains one of the most concerning complications [6,7]. Cisp exposure has been shown to cause testicular atrophy, along with marked reductions in sperm count, viability, and motility, ultimately impairing spermatogenesis and leading to infertility [8]. This damage is directly related to oxidative stress, as indicated by higher levels of malondialdehyde (MDA) and nitric oxide (NO), alongside depleted concentrations of key antioxidants such as superoxide dismutase (SOD), glutathione (GSH), and catalase (CAT) in testicular tissue [9]. In addition, Cisp has been found to suppress serum levels of testosterone, luteinizing hormone (LH), and follicle-stimulating hormone (FSH) [10], while simultaneously increasing inflammatory markers including MDA, NO, nuclear factor kappa B (NF-κB), and tumor necrosis factor alpha (TNF-α) in the testes [11]. Ferroptosis is an iron-dependent type of controlled cell death characterized by lipid peroxidation and the failure of cellular antioxidant defenses, specifically glutathione peroxidase 4 (GPX4) activity. Evidence suggests that chemotherapeutics, such as Cisp, can activate ferroptotic signaling in sensitive tissues, adding to cumulative toxic effects [12]. Despite significant evidence of its toxic effects, the precise mechanisms behind Cisp-induced testicular damage are still not entirely known. As a result, targeting ferroptosis—whether by restricting iron-mediated processes or restoring antioxidant systems—represents an intriguing method to minimize Cisp-associated tissue damage [13].

*Vitis vinifera* L., a perennial vine in the *Vitaceae* family, is commonly grown for grape and wine production. Its seeds are a great source of oil that contains essential fatty acids, especially linoleic acid (65–75%)—as well as vitamin E, phytosterols, and various phenolic constituents such as catechins, epicatechins, and gallic acid. These bioactive compounds give grape seed oil strong nutritional and therapeutic potential, supported by both in vitro and a growing number of in vivo studies. Regular consumption has been associated with enhanced antioxidant enzyme activity, reduced inflammation, anti-atherosclerosis effects, and defense against cellular oxidative damage and certain malignancies [14]. Grape seeds are especially notable for their high content of proanthocyanidin oligomers, plant-derived flavonoids recognized for their potent antioxidant capacity [15]. *Vitis vinifera* L. extract, concentrated from these seeds, contains 70–95% proanthocyanidins, regarded as the extract’s most bioactive constituents [16]. *Vitis vinifera* L. is widely studied for its strong free radical scavenging properties and has shown promise in counteracting the toxic effects of various xenobiotics. Of relevance is the *Vitis vinifera* L. extract’s potential to mitigate Cisp-induced toxicity. Cisp, though an effective chemotherapeutic, is known to cause oxidative stress, inflammation, and apoptosis in testicular tissue. Due to its antioxidant, anti-inflammatory, anti-apoptotic, and anticarcinogenic activities, *Vitis vinifera* L. extract has been explored as a protective agent against such damage [17]. Experimental evidence supports its use in preventing organ damage, including cardiotoxicity, neurotoxicity [18], nephrotoxicity [19], and metabolic disturbances [20]. Mechanistically, *Vitis vinifera* L. extract has been found to suppress the generation of ROS by inhibiting specific enzyme systems. It also exhibits antimutagenic and anticarcinogenic effects [21,22], likely through modulation of key signaling pathways. *Vitis vinifera* L. enhances cellular defense by up-regulating phase II detoxification enzymes via activation of nuclear factor erythroid 2–related factor 2 (NRF2) [23], down-regulating inflammatory mediators like NF-κB and protein kinase C (PKC) [24], and modulating apoptosis-related proteins, including caspase-1, iNOS, calpain-1, caspase-3, and B-cell lymphoma 2 (Bcl-2) [25]. Despite these findings, the precise mechanisms by which *Vitis vinifera* L. extract protects against Cisp-induced testicular apoptosis remain to be fully elucidated. This is similarly the case for *Vitis vinifera* L. extract’s capacity to stabilize capillary membranes, increase intracellular vitamin C, and neutralize oxidative stress [26,27]. Proanthocyanidin oligomers exhibit antioxidant activity that is estimated to be nearly 50 times as powerful as vitamins E and C [28]. However, the capacity of *Vitis vinifera* L. extract to counteract Cisp-induced testicular injury, specifically through the modulation of ferroptotic mechanisms, has not been fully elucidated. The purpose of this work was to investigate the preventive efficacy of *Vitis vinifera* L. extract in reducing Cisp-induced oxidative damage and reproductive impairment in rat testes, as well as to explain the molecular processes underlying these effects.

## 2. Results

### 2.1. Phytochemical Characterization of Vitis vinifera Seed Extract

**Figure 1:**
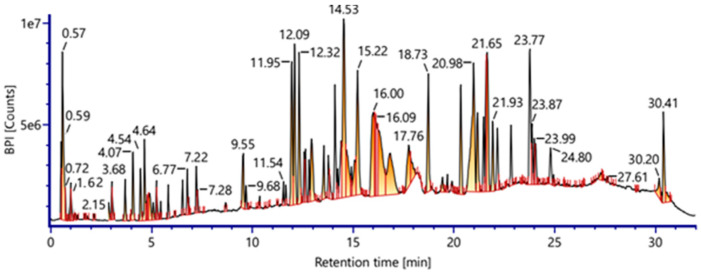
Total ion chromatogram of QTOF-HRMS/MS analysis (negative mode) of *Vitis vinifera* seeds extract.

**Figure 2:**
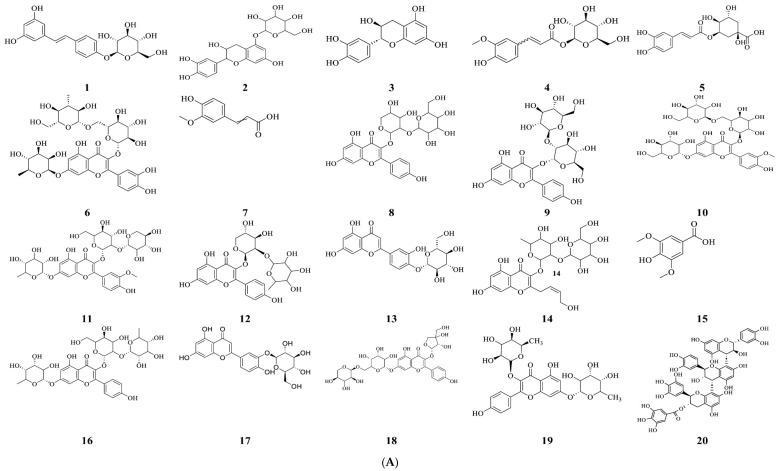

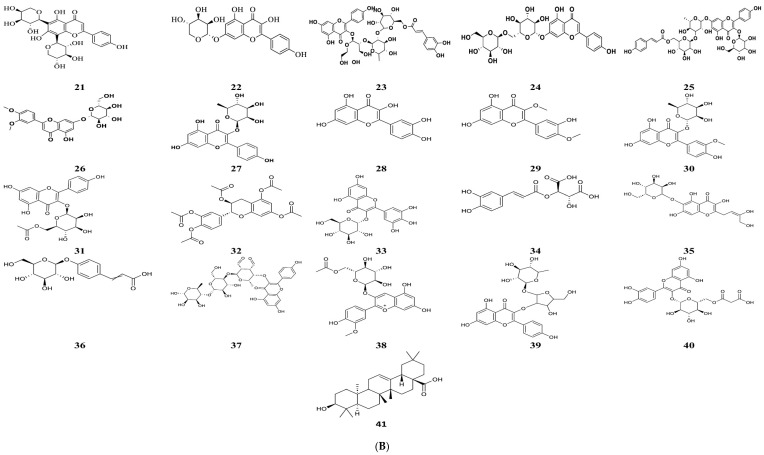
(**A**) Chemical structures of the most abundant compounds (**1**–**20**) detected in negative modes of the QTOF-HRMS/MS analysis of *Vitis vinifera* seeds extract. (**B**) Chemical structures of the most abundant compounds (**21**–**41**) detected in negative modes of the QTOF-HRMS/MS analysis of *Vitis vinifera* seeds extract.

Using the negative mode of QTOF-HRMS/MS, 41 chemicals were tentatively identified in a *Vitis vinifera* L. seed extract. The primary components have distinct chemical ontologies, such as phenolic acids, flavonoids, proanthocyanidins, terpenoids, and other glycosylated molecules. Although the extract was not standardized to a specific bioactive marker, reproducibility was supported by generating a detailed QTOF-HRMS/MS–based metabolomic fingerprint that documents the major and minor constituents of the Vitis seed extract. Table 1 and Figure 1 show the metabolite profiles that were obtained. The chemical structures of the most abundant compounds detected in the *Vitis vinifera* L. seed extract by QTOF-HRMS/MS in negative mode are shown in Figure 2A,B.

### 2.2. The Effect of Vitis vinifera Extract on Blood FSH, LH, and Testosterone Levels in Cisplatin-Induced Testicular Damage in Rats

**Figure 3:**
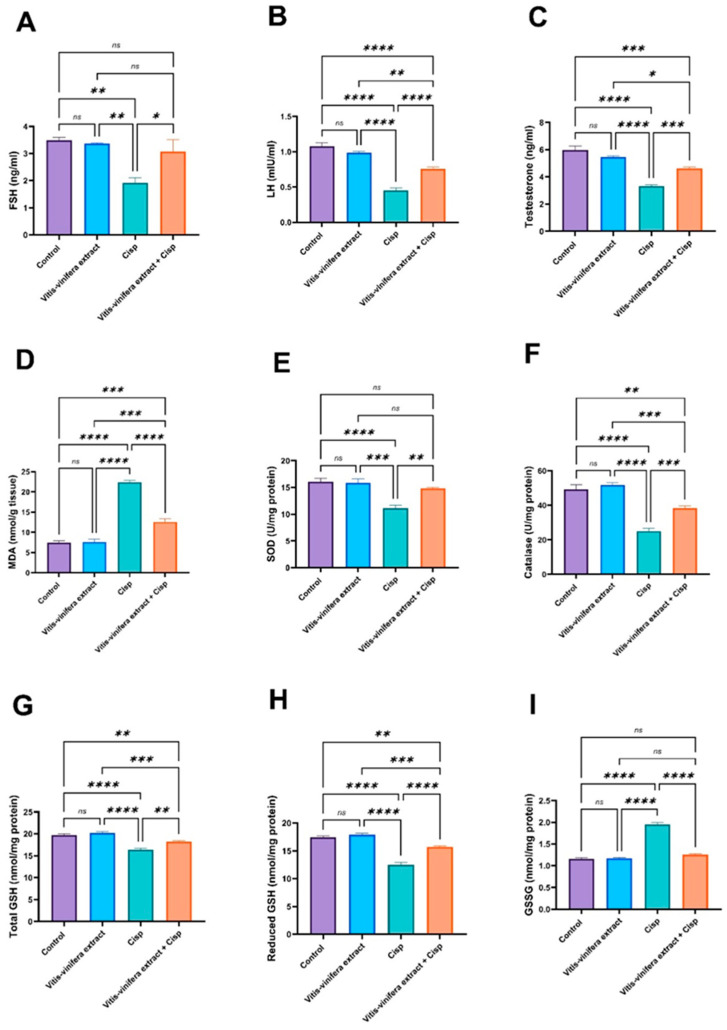
Effect of *Vitis vinifera* extract treatment on (**A**) follicle-stimulating hormone (FSH), (**B**) luteinizing hormone (LH), (**C**) testosterone level, (**D**) malondialdehyde (MDA), (**E**) superoxide dismutase (SOD), (**F**) catalase, (**G**) total glutathione (Total GSH), (**H**) reduced glutathione (reduced GSH), and (**I**) oxidized glutathione (GSSG) levels against Cisplatin (Cis)-induced testicular toxicity in a rat model. Tukey’s test was used to examine the data after a one-way ANOVA, and the mean ± SE (n = 5) was used to express the result that was obtained. The following symbols indicate statistical significance: ns = nonsignificant, * = *p* < 0.05, ** = *p* < 0.01, *** = *p* < 0.001, and **** = *p* < 0.0001.

As indicated in Figure 3A–C, serum FSH, LH, and testosterone levels did not show any significant change between those parameters in the Control and *Vitis vinifera* L. extract groups. On the contrary, Cisp-intoxicated rats showed a significant (*p* < 0.05) decrease in serum FSH, LH, and testosterone levels (by 44.99, 58.07, and 44.37%, respectively) compared to the control group. In contrast, the co-treated (*Vitis vinifera* L. extract + Cisp.) group had significantly (*p* < 0.05) higher serum FSH, LH, and testosterone levels (by 59.83, 66.81, and 38.60%) relative to the Cisp-intoxicated rats.

### 2.3. Effect of Vitis vinifera Extract on the Testicular Oxidative/Anti-Oxidative Biomarkers (MDA, SOD, CAT, Total GSH, Reduced GSH, GSSG, NRF2, Ho-1, Gpx4, and 8-OHDG) in Cisplatin-Induced Testicular Damage in Rats

The Cisp-intoxicated group demonstrated a considerable (*p* < 0.05) increase in testicular malonaldehyde (MDA) (by 199.60%) activity as compared to the control group. In addition, superoxide dismutase (SOD) and catalase (CAT) activities were significantly (*p* < 0.05) reduced (by 31.02% and 49.07%) when compared to the control group. In contrast, the co-treatment considerably lowered the MDA level by 43.95%. Also, there was a considerable (*p* < 0.05) up-regulation in SOD and CAT activities by 33.09% and 52.99%, respectively, in comparison to the Cisp-intoxicated group (Figure 3D–F). Testicular total glutathione (tGSH), reduced glutathione (GSH), and oxidized glutathione (GSSG) levels did not differ significantly between the *Vitis vinifera* L.-treated and control groups. Conversely, Cisp administration induced a significant (*p* < 0.05) disruption of glutathione redox balance, as evidenced by a marked depletion of total and reduced GSH (by 17.15% and 28.48%, respectively) and a pronounced alteration in GSSG levels (by 68.51%) compared with controls. Notably, co-treatment with *Vitis vinifera* L. extract significantly (*p* < 0.05) restored glutathione homeostasis, reflected by an increase in total and reduced GSH levels (by 11.1% and 25.72%) and a concomitant reduction in GSSG levels (by 35.62%) relative to Cisp-intoxicated rats, as shown in Figure 3G–I.

**Figure 4:**
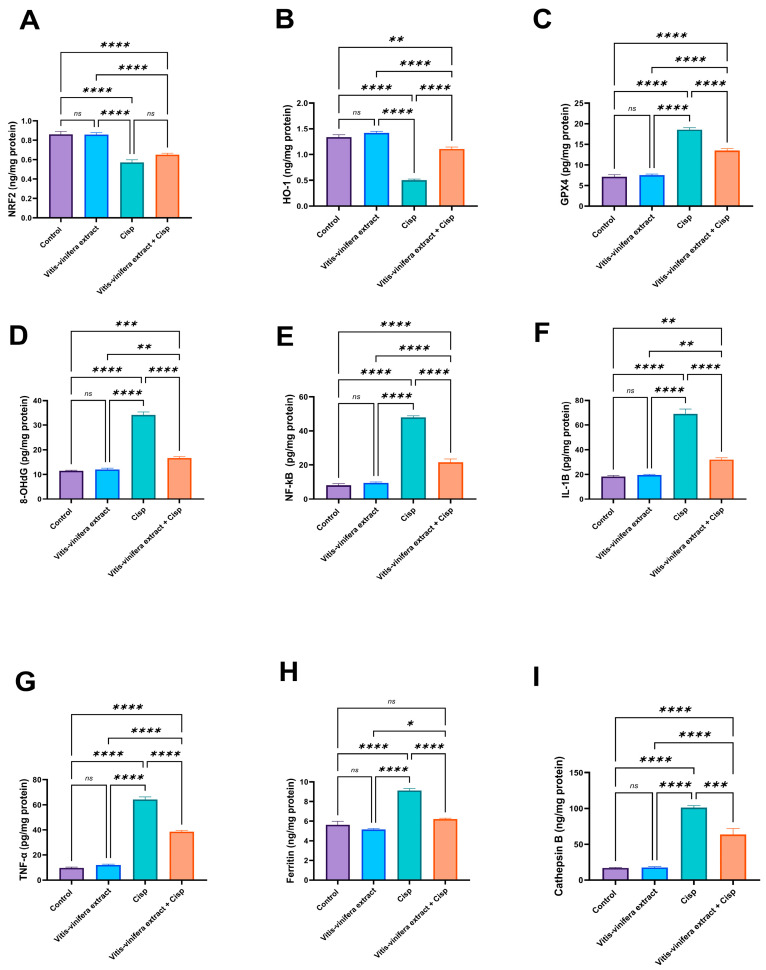
Effect of *Vitis vinifera* extract treatment on the protein levels of (**A**) nuclear factor erythroid2-related factor2 (NRF2), (**B**) heme Oxygenase-1 (HO-1), (**C**) glutathione peroxidase 4 (GPX4), (**D**) 8-Hydroxy-2′-deoxyguanosine (8-OHdG), (**E**) nuclear factor kappa B (NF-KB), (**F**) interleukin (IL)-1B, (**G**) tumor necrosis factor alpha (TNF)-α level, (**H**) ferritin, and (**I**) Cathepsin B levels against Cisplatin (Cisp)-induced testicular toxicity in a rat model. Tukey’s test was used to examine the data after a one-way ANOVA, and the mean ± SE (n = 5) was used to express the result that was obtained. The following symbols indicate statistical significance: ns = nonsignificant, * = *p* < 0.05, ** = *p* < 0.01, *** = *p* < 0.001, and **** = *p* < 0.0001.

In addition, there were no significant differences in the levels of nuclear factor erythroid 2–related factor 2 (NRF2), heme Oxygenase-1 (HO-1), and 8-hydroxy-2′-deoxyguanosine (8-OHDG) between the *Vitis vinifera* L. extract-treated and control groups, whereas the Cisp group exhibited a significant (*p* < 0.05) 33.48% and 62.28% decrease in NRF2 and HO-1 levels, respectively, with a noticeable (*p* < 0.05) increase in glutathione peroxidase 4 (Gpx4) (by 22.7%) and 8-OHDG level (by 197.90%), compared to the control. In contrast, the co-treated group showed no significant change in NRF2 levels compared to the cis-intoxicated group. However, a significant (*p* < 0.05) increase in Ho-1 level (by 119.84%) and decrease in Gpx4 and 8-OHDG levels (by 27.4% and 51.03%, respectively) when compared to Cisp-intoxicated rats is shown in Figure 4A–D.

### 2.4. Effect of Vitis vinifera Extract on Testicular Pro-Inflammatory Cytokines as NFκB IL-1β, TNF-α and Some Ferroptosis Factors, in Cisplatin-Induced Testicular Damage in Rats

According to Figure 4E–G, there were no significant variations in NFκB, IL-1ẞ, and TNF-α levels between the *Vitis vinifera* L. extract and control groups. Cisp treatment substantially (*p* < 0.05) increased those biomarkers by 493.88%, 276.83%, and 559.51%, respectively, as compared to the control group. Co-treatment with *Vitis vinifera* L. extract and Cisp resulted in significant (*p* < 0.05) down-regulation of those biomarkers by 55.08%, 53.51%, and 39.98%, respectively, compared to the Cisp-intoxicated group. Furthermore, there were nonsignificant variations in ferritin and cathepsin levels between the *Vitis vinifera* L. extract-treated group and the control group, but both parameters showed marked (*p* < 0.05) up-regulation (by 61.85% and 498.34%) in the Cisp-intoxicated group compared to the control one. When compared to the Cisp-treated group, their levels in the co-treated group were significantly (*p* < 0.05) lower (31.94% and 37.19%) (Figure 4H,I).

### 2.5. Effect of Vitis vinifera Extract on Gene Expression of ARNTL, ASCL4, PI3K, GSK3B, COX2, and miRNA 125-b in Cisplatin-Induced Testicular Damage in Rats

**Figure 5:**
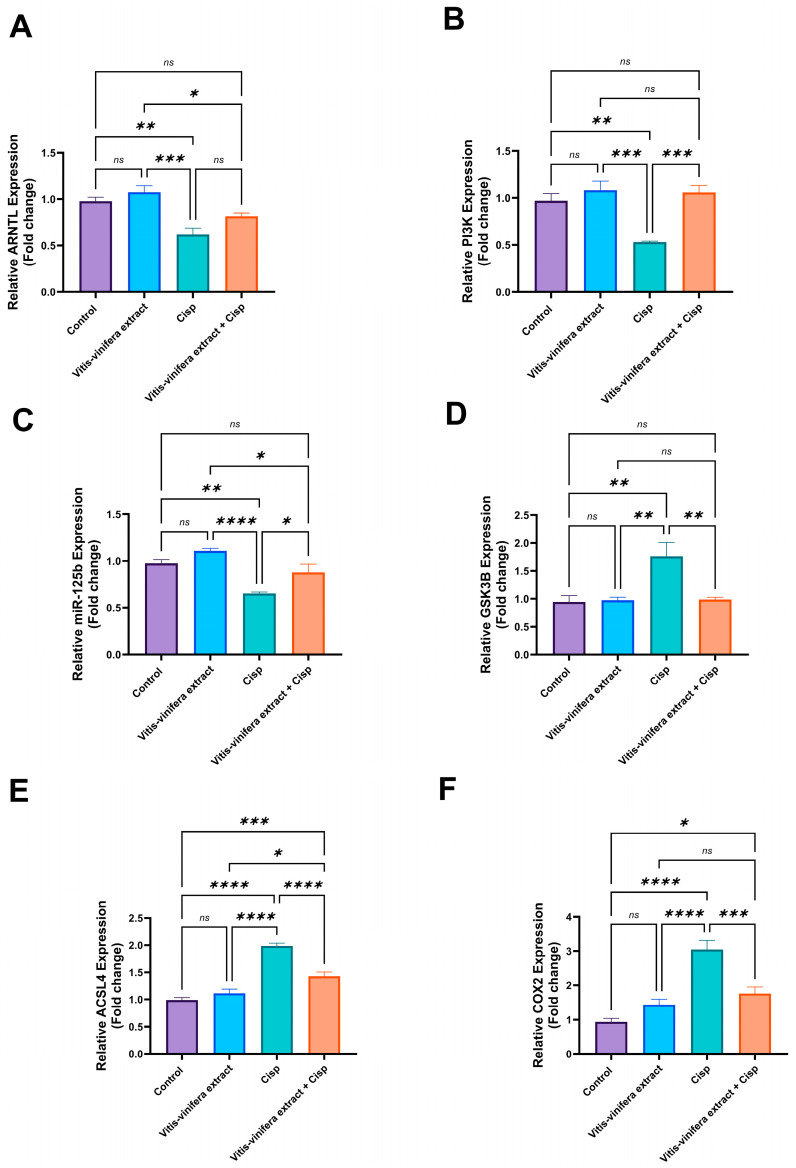
Effect of *Vitis vinifera* L. extract treatment on (**A**) Aryl Hydrocarbon Receptor Nuclear Translocator-Like/BMAL1 (ARNTL) gene expression, (**B**) Phosphoinositide 3-Kinase (PI3K) gene expression, (**C**) miR-125b, (**D**) Glycogen Synthase Kinase 3 Beta (GSK3B) gene expression, (**E**) Acyl-CoA Synthetase Long-Chain Family Member 4 (ACSL4) gene expression, and (**F**) Cyclooxygenase-2 (COX2) gene expression against cisplatin (Cisp)-induced testicular toxicity in a rat model. Tukey’s test was used to examine the data after a one-way ANOVA, and the mean ± SE (n = 5) was used to express the result that was obtained. The following symbols indicate statistical significance: ns = nonsignificant, * = *p* < 0.05, ** = *p* < 0.01, *** = *p* < 0.001, and **** = *p* < 0.0001.

Figure 5A–C show that Cisp significantly (*p* < 0.05) down-regulated the gene expression of Aryl Hydrocarbon Receptor Nuclear Translocator-Like (ARNTL), Phosphoinositide 3-Kina (PI3K), and MicroRNA-125b (miRNA 125-b) in testicular tissue by 36.6%, 45.36%, and 33.05%, respectively, when compared to the control group. Treatment with *Vitis vinifera* L. extract significantly (*p* < 0.05) increased the gene expression of PI3K and miRNA 125-b by 99.6% and 34.6%, respectively, compared to the Cisp group. However, the gene expression of ARNTL nonsignificantly increased compared to the Cisp group. Furthermore, there were nonsignificant variations in those parameters levels between the *Vitis vinifera* L. extract-treated group and the control group.

On the other hand, Cisp significantly (*p* < 0.05) up-regulated the gene expression of GSK3B, ASCL4, and COX2 in testis tissue by 86.8%, 100.6%, and 224.9%, respectively, when compared to the control group. Treatment with *Vitis vinifera* L. extract significantly (*p* < 0.05) decreased the gene expression of GSK3B, ASCL4, and COX2 in testis tissue by 44%, 27.9%, and 42.3%, respectively. The *Vitis vinifera* L. extract control group showed a nonsignificant change from the control group in those genes (Figure 5D–F).

### 2.6. Effect of Vitis vinifera L. Extract on Sperm Count and Motility in Cisplatin-Induced Testicular Injury in Rats

**Figure 6:**
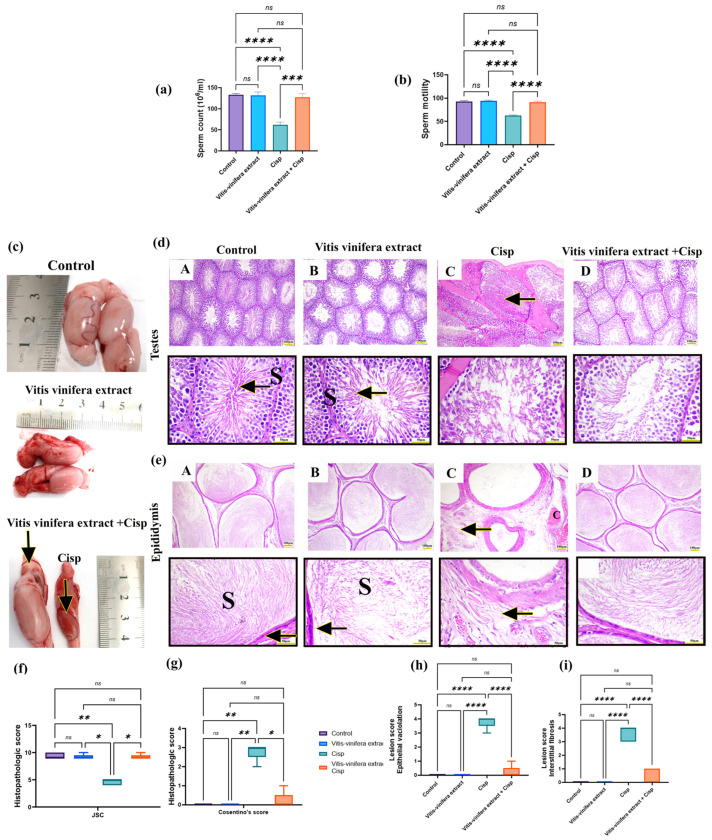
Effect of *Vitis vinifera* L. extract treatment on sperm count and motility in cisplatin (Cisp)-induced testicular toxicity in rat model. (**a**) Sperm count. (**b**) Sperm motility. (**c**) Macroscopic picture of testes from different experimental groups (arrows indicate atrophied, congested testicles in the Cisp group relative to nearly normal testis in the combination group). (**d**) Hematoxylin and eosin (H&E) stained testes sections, (**A**): control group and (**B**): *Vitis vinifera* L. extract group showing normal seminiferous tubule with lining spermatogonia (S) and spermatozoa (arrow), (**C**): Cisp group showing degeneration and disorganization of germinal epithelium, depletion and desquamation of germ cells into lumen with hyalinization (arrow), (**D**): *Vitis vinifera* L. extract + Cisp group showing restoration of testicular structure. (**e**) Hematoxylin and eosin (H&E) stained epididymal sections, (**A**): control group and (**B**): *Vitis vinifera* extract group showing normal epithelial lining (arrow) with numerous intraluminal sperms (S), (**C**): Cisp group showing congested vessels (C) with interstitial fibrosis (arrow), (**D**): *Vitis vinifera* L. extract + Cisp group showing restoration of epididymal structure. Bar (**d**(**A**–**D**),**e**(**A**–**D**)) = 100 µm and squared boxes = 50 µm. (**f**) Johnson spermatogenic histologic score (JSS). (**g**) Cosentino’s score. (**h**) Epididymal epithelial vacuolation score. (**i**) Interstitial fibrosis score. Mean ± SE (n = 5) was used to express the result that was obtained. The following symbols indicate statistical significance: ns = nonsignificant, * = *p* < 0.05, ** = *p* < 0.01, *** = *p* < 0.001, and **** = *p* < 0.0001.

The Cisp-treated group had a significant (*p* < 0.05) decrease in sperm count and progressive sperm motility of 53.31 and 32.54%, respectively, compared to the control group. In contrast, rats treated with Cisp along with *Vitis vinifera* L. extract showed a substantial (*p* < 0.05) increase in sperm count and progressive sperm motility of 104.52% and 45.65%, respectively, compared to the Cisp-treated group. However, there were no significant differences in sperm characteristics between *Vitis vinifera* L. extract and control rats (Figure 6a,b).

### 2.7. Effect of Vitis vinifera L. Extract on Testicular and Epididymal Histoarchitecture in Cisplatin-Induced Testicular Injury in Rats

Macroscopic picture of control and *Vitis vinifera* L. extract showed nearly normal testicular and epididymal tissue. At the same time, Cisp-intoxicated rates showed atrophied, congested, and firm texture of both testicles and epididymis. Conversely, the *Vitis vinifera* L. extract + Cisp-treated group showed restoration of testicular and epididymal tissues (Figure 6c).

Rat testes in the control and *Vitis vinifera* L. extract-treated groups showed characteristic interstitial Leydig cells and normal-sized seminiferous tubules with different spermatozoa growth stages when examined under a microscope (Figure 6(dA) and Figure 6f, respectively). Rats given Cisp, on the other hand, had vascular congestion, degeneration and disorganization of germinal epithelium, and depletion and desquamation of germ cells into the lumen with hyalinization of it content (Figure 6(dC)). Additionally, the *Vitis vinifera* L. extract + Cisp-treated group showed enormous intraluminal spermatozoa and improved testicular histoarchitecture (Figure 6(dD)). Rat epididymal tissue in the control and *Vitis vinifera* L. extract-treated groups showed typical ciliated cuboidal epithelium with massive intraluminal spermatozoa (Figure 6(eA) and Figure 6(eB), respectively). Rats given Cisp, on the other hand, had vascular congestion, epithelial vacuolization with interstitial fibrosis, and empty tubules (Figure 6(eC)). Additionally, the *Vitis vinifera* L. extract + Cisp-treated group showed enormous intraluminal spermatozoa and restoration of epididymal epithelial histoarchitecture (Figure 6(eD)).

Statistical analysis of Johnsen’s histologic grading showed that Cisp-treated animals had a considerable (*p* < 0.05) decrease in Johnsen’s score relative to control and *Vitis vinifera* L. extract groups. Furthermore, this score was substantially (*p* < 0.05) up-regulated in the testicles of the *Vitis vinifera* L. extract + Cisp group, equated to the Cisp-only group (Figure 6f). Cosentino’s score revealed a significant (*p* < 0.05) increase in degenerative alterations in Cisp-only-treated animals compared to the control and *Vitis vinifera* L. extract groups. However, the Vitis vinifera L. extract + Cisp group demonstrated a significant (*p* < 0.05) decrease in Cosentino’s score when compared to Cisp-treated animals (Figure 6g). Epididymal lesion scores (Vacuolation and interstitial fibrosis) significantly (*p* < 0.05) increased relative to control and *Vitis vinifera* L. extract rats. At the same time, the *Vitis vinifera* L. extract + Cisp-treated group exhibited a significant (*p* < 0.05) down-regulation of this score relative to the Cisp group (Figure 6h,i).

### 2.8. Effect of Vitis vinifera L. Extract on NF-kB p65 and PCNA Immune-Expression in Cisplatin-Induced Testicular and Epididymal Damage in Rats

**Figure 7:**
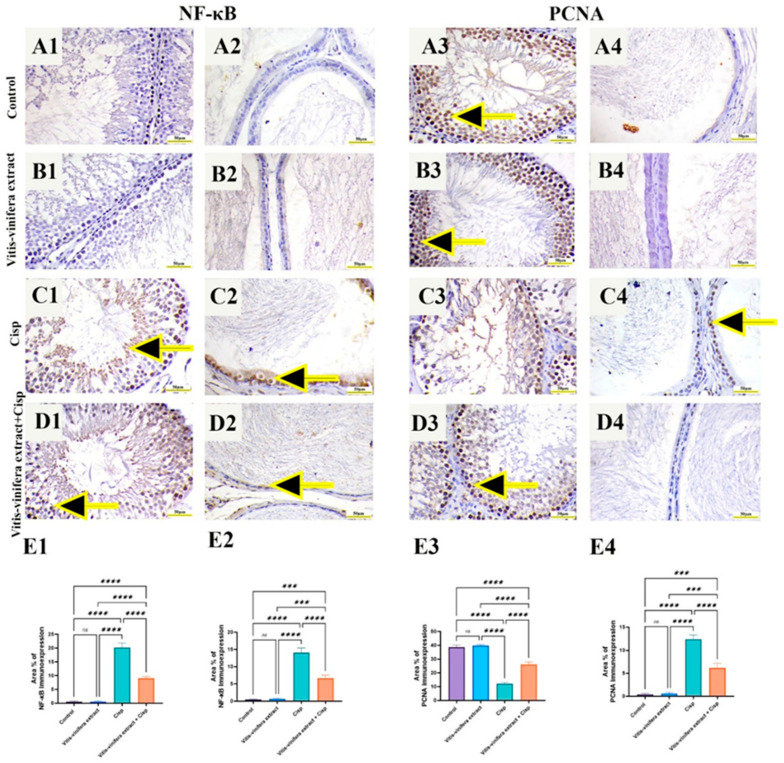
Effect of *Vitis vinifera* L. extract treatment on immunohistochemical expression of nuclear factor kappa beta (NF-kB p65) and proliferating cell nuclear antigen (PCNA) in Cisplatin (Cisp)-induced rat testicular toxicity. (**A1**–**D1**) NF-kB p65 expression in testicular tissue of different experimental groups. (**E1**) Area % of testicular NF-kB p65 staining. (**A2**–**D2**) NF-kB p65 expression in epididymal tissue of different experimental groups. (**E2**) Area % of epididymal NF-kB p65 staining. (**A3**–**D3**) PCNA expression in testicular tissue of different experimental groups. (**E3**) Area % of testicular PCNA staining. (**A4**–**D4**) PCNA expression in epididymal epithelium of different experimental groups. (**E4**) Area % of epididymal PCNA staining. Arrows indicate positive brown nuclear immunostaining. Bar = 50 µm. Tukey’s test was used to examine the data after a one-way ANOVA, and the mean ± SE (n = 5) was used to express the result that was obtained. The following symbols indicate statistical significance: ns = nonsignificant, *** = *p* < 0.001, and **** = *p* < 0.0001.

NF-κB immunoreactivity in several experimental groups is shown in Figure 7. Rat testicular (Figure 7(A1,B1)) and epididymal (Figure 7(A2,B2)) NF-κB immunoreactivity showed that both the control and *Vitis vinifera* extract had negative nuclear immunoreactivity. In contrast to control rats, Cisp-treated rats displayed a high level of nuclear immunoreactivity in both testicular and epididymal tissue (Figure 7(C1,C2)). The *Vitis vinifera* L. extract + Cisp group’s NF-κB immunoreactivity ranged from mild immunoreactivity in both testicular and epididymal tissue (Figure 7(D1,D2)). When compared to the control and *Vitis vinifera* extract-treated groups, the Cisp-treated group’s NF-κB immunoreactivity area percentage was significantly (*p* < 0.05) higher in both testicular and epididymal tissue. In contrast to animals treated with Cisp, the *Vitis vinifera* L. extract + Cisp group displayed a significant decline in NF-κB immunoreactivity area% in both testicular and epididymal tissue (Figure 7(E1,E2)).

Proliferating Cell Nuclear Antigen (PCNA) immunoreactivity in several experimental groups is illustrated in Figure 7. Rat testicular PCNA immunoreactivity showed that both the control and *Vitis vinifera* L. extract had intense nuclear immunoreactivity in testes (Figure 7(A3,B3)) and negative immunoreactivity in epididymal epithelium (Figure 7(A4,B4)). In contrast to control rats, Cisp-treated rats displayed a low level of nuclear immunoreactivity in testes (Figure 7(C3)) and moderate-to-intense immunoreactivity in epididymal epithelium (Figure 7(C4)). The *Vitis vinifera* L. extract + Cisp group’s PCNA immunoreactivity showed moderate immunoreactivity in testes (Figure 7(D3)) and mild immunoreactivity in epididymal epithelium (Figure 7(D4)). When compared to the control and *Vitis vinifera* extract-treated groups, the Cisp-treated group’s PCNA immunoreactivity area percentage was significantly lower in testes and higher in epididymis. In contrast to animals treated with Cisp, the *Vitis vinifera* L. extract + Cisp group displayed a significant (*p* < 0.05) elevation in PCNA immunoreactivity area% in testes and a decrease in epididymal epithelium (Figure 7(E3,E4)).

## 3. Discussion

Cisp is a commonly used antineoplastic agent against many types of cancers, but its use is correlated with noteworthy testicular intoxication because of its vigorous cytotoxic action. The process of spermatogenesis is extremely affected as the chemotherapy drugs always target the dividing cells. Various studies have reported that Cisp impairs the reproductive function [29]. It was reported that Cisp leads to testicular disintegration, apoptosis of germ cells, sperm dysfunction, and Leydig cells membrane rigidity and other abnormalities, also it can result in oxidative damage and lipid peroxidation and a decline in antioxidants. Cisp causes increased amounts of both ROS and RNS, resulting in oxidative stress [30]. *Vitis vinifera* L. extract is considered one of the most effective natural antioxidants. Many studies reported that the potent antioxidant and anti-apoptotic effects of *Vitis vinifera* L. extract may be the main cause of its protective impacts on male fertility [31]. Therefore, the current study aimed to investigate the potential therapeutic effects of *Vitis vinifera* L. extract against Cisp-induced testicular injury in adult male rats.

Our study found that Cisp administration affected male rats’ fertility directly. That was confirmed by a notable decline in serum FSH, LH, and testosterone levels related to a decrease in sperm motility and number. This may be attributed to the inhibitory effect of Cisp on testosterone synthesis via suppression of 17-α-hydroxylase enzyme or reducing the receptors of LH on Leydig cells, as reported by Ibrahim et al. [32]. Also, our results harmonized with those of Tian, Liu, Liu, Zhang, Li, Hou, Zhao, Li, Chang and Sun [17] who reported that FSH, LH, and testosterone levels in serum were reduced after a single injection of Cisp (10 mg/kg I/P). Furthermore, in line with our findings, Mahran et al. [33] found that Cisp lowered LH levels and serum testosterone by inhibiting steroidogenic enzymes. But our results are in contrast with Heibashy and Morsy [34], who found that the administration of Cisp induced substantial increments in the serum levels of both FSH and LH with a significant decrease in the level of testosterone; their findings could be attributed to Cisp’s ability to alter pituitary sensitivity to gonadotropin-releasing hormone (GnRH) stimulation, as opposed to hypothalamic–pituitary–adrenal axis dysfunctions and/or excess adrenocortical product perfusion and response to adrenocorticotropic hormone (ACTH) motivation. Furthermore, higher levels of FSH and LH may be due to a defect in androgen-mediated negative feedback regulation of gonadotropins. *Vitis vinifera* L. extract, on the other hand, resulted in significant changes in serum FSH, LH, and testosterone levels. Our findings align with Mady and Sawires [35], who showed a considerable up-regulation in the testosterone levels after co-administration of *Vitis vinifera* L. extract and AL2O3-NPs. This elevation was discussed by other researchers who found that *Vitis vinifera* L. extract can alleviate the inhibitory action of Cisp on interstitial Leydig cells through elevating the expression of mRNA of the testosterone synthetase; this thus retrieves the suppression of testosterone synthesis by up-regulation of the expression of the essential enzymes accountable for testosterone synthesis, according to Tian et al. [36].

Our study reported that Cisp administration to male rats substantially elevated testicular oxidative stress. Results revealed an up-regulation in the testicular MDA and GSSG and 8-OHDG levels and a decline in total and reduced GSH levels, CAT, SOD, and NRF2-2/HO-1 activities. Consistent with our findings Tozak Yıldız, Kalkan, Baydilli, Gönen, Cengiz Mat, Köseoğlu, Önder and Yay [29] reported in an earlier study that the Cisp group revealed a significant decline in GSH-PX, SOD, and CAT activities in testicular homogenate and increased lipid peroxidation metabolite (MDA) level, in comparison to the control one. This may be ascribed to the mitochondrial damage by Cisp, resulting in an increase in ROS and oxidative stress [36]. Our results come in the same line with Tian, Gao, Rudebush, Yu and Zucker [36] who indicated that Cisp decreased both SOD and GSH-Px activities. In addition, MDA and 8-OHdG levels were elevated in rat testes. Conversely, our study exhibited that *Vitis vinifera* L. extract counteracted the oxidative stress damage induced by Cisp, as it substantially antagonized all the above-mentioned toxic effects induced by Cisp. Our findings were consistent with Tian, Gao, Rudebush, Yu and Zucker [36], who found that *Vitis vinifera* L. extract might protect testes from oxidative damage caused by Cisp. Furthermore, Mady and Sawires [35] reported that *Vitis vinifera* L. extract reduced the lethal effect of cadmium-induced testicular injury by attenuating oxidative stress due to its significant antioxidant action and being a robust free radical scavenger. They also observed that administering *Vitis vinifera* L. extract activated the NRF2 pathway by significantly increasing NRF2 and HO-1, resulting in decreased oxidative indicators and increased antioxidant enzymes [35]. Exposure to high levels of Cisp is the primary cause of massive ROS generation. Cisp acts by depleting sulfhydryl (SH) group-containing substances such as glutathione (GSH), which is then transformed to a thiol radical and creates ROS from molecular oxygen. *Vitis vinifera* L. extract possesses strong antioxidant and free radical scavenging properties. Also, it has significantly higher antioxidant activity than vitamins C, E, and carotenes [17].

Our study revealed that Cisp administration to male rats significantly induced testicular apoptosis and inflammation, as evidenced by the up-regulation of TNF, IL-1ẞ, and NFκB. An earlier study by Heibashy and Morsy [34] supported our findings; both authors indicated that Cisp induced inflammation and increased apoptosis, as evidenced by increasing TNF-α and NFκB pathways. Demir et al. [37] also indicated that Cisp accelerated inflammatory cell damage via activating the NF-κB pathway and by up-regulating the levels of pro-inflammatory cytokines, involving interleukin-6 (IL-6). The anti-inflammatory effects observed can be partly attributed to the modulation of upstream TLR–NF-κB signaling. Oxidative stress is a well-recognized activator of Toll-like receptors, particularly TLR4, which promotes NF-κB nuclear translocation and subsequent transcription of pro-inflammatory cytokines. Accordingly, attenuation of oxidative stress is expected to suppress TLR activation and downstream NF-κB signaling [38,39]. Concretely, our work demonstrated that *Vitis vinifera* extract ameliorated the inflammation induced by Cisp via suppression of TNF, IL-1ẞ, and NF-κB in testicular tissues.

Our work indicated that Cisp administration to male rats markedly induced testicular ferroptosis, evidenced by up-regulation of ferritin and cathepsin. Similarly, Mahran et al. [33] found that Cisp induced ferroptosis, evidenced by iron overload in the testes and altered the mRNA expression of genes involved in regulating ferroptosis, such as iron uptake, antioxidant defense, and lipid metabolism. Conversely, our findings revealed that *Vitis vinifera* extract can attenuate ferroptosis and alleviate testicular damage induced by Cisp via activation of the NRF2 pathway and down-regulation of ferritin and cathepsin. Our results were in agreement with Li et al. [40] who reported that *Vitis vinifera* extract can treat ferroptosis as it activates the Nrf2 pathway, the antagonist of ferroptosis. *Vitis vinifera* extract is a natural regulator of iron metabolism and can treat iron overload diseases [41]. Another study has revealed that polyphenols such as *Vitis vinifera* extract act as iron chelators and suppress GSH depletion and lipid peroxidation, and protect murine MIN6 pancreatic cells against iron toxicity and erastin-induced ferroptosis [40].

Cisp-induced ferroptosis is evidenced by iron overload in the testis, and this was described in previous work of Cisp-induced testicular injury [42], nephrotoxicity [43], and ototoxicity [44]. Additionally, this study showed that Cisp up-regulated the gene expression of ACSL4, which encodes for acyl-CoA synthetase long-chain family member 4. It is a lipid-metabolism enzyme that dictates susceptibility to ferroptosis, the iron-dependent, lipid-peroxidation form of regulated cell death. Increased ACSL4 expression is a canonical pro-ferroptotic signature, while reduced GPX4 is often the countervailing anti-ferroptotic marker reported in male reproductive cells [33]. Recent studies link ferroptosis to testicular injury and sperm dysfunction reported increases in ACSL4, documented in the testis under oxidative stress [45,46]. Thus, the increased ACSL4 observed here is consistent with ferroptotic stress contributing to Cisp toxicity. Our findings were in line with those obtained by Mahran, Badr, Aloyouni, Alkahtani, Sarawi, Ali, Alsultan, Almufadhili, Almasud and Hasan [33].

However, when *Vitis vinifera* extract was given alongside Cisp, it significantly decreased the expression of the ferroptosis gene ACSL4 than the Cisp group. This suggested that this extract might prevent Cisp-induced ferroptosis, which is one of the main mechanisms causing testicular injury. These results were in agreement with Lv et al. [47], who reported that proanthocyanidin from *Vitis vinifera* extract reduced ferroptosis by up-regulating GSH and GPX and lowering ACSL4 in mice with acute lung damage. To the best of our knowledge, this investigation was the first to identify the possible protective effect of *Vitis vinifera* extract on testicular damage by Cisp through targeting the ferroptosis pathway, providing new insights into the involvement of this extract in ferroptosis control.

ARNTL (also called BMAL1) is a core circadian transcription factor that is essential for normal testicular function [48]. In the current study, Cisp significantly down-regulated the gene expression of ARNTL in testis tissue compared to the control group. It was reported by Alvarez, Hansen, Ord, Bebas, Chappell, Giebultowicz, Williams, Moss and Sehgal [48] that ARNTL loss impairs Leydig cell steroidogenesis, decreases testosterone, disrupts spermatogenesis and fertility, and makes testicular cells more vulnerable to oxidative-stress-linked damage and impaired recovery. Furthermore, genetic analyses suggest that polymorphisms in circadian clock genes like ARNTL may be associated with idiopathic male infertility [49]. ARNTL has a protective effect against ferroptosis-mediated inflammation and ensures expression of crucial antioxidant and membrane repair genes—such as SLC7A11, GPX4, SOD1, thus curbing ferroptotic tissue damage in experimentally induced pancreatitis in mice [50]. These findings suggest a possible link between ARNTL gene down-regulation and testicular dysfunction induced by Cisp. In this study, when *Vitis vinifera* extract was given alongside Cisp, it significantly increased the expression of the ARNTL gene and ameliorated the increase in oxidative stress and ferroptosis in Cisp-induced testicular damage. These results were in agreement with Rodríguez et al. [51] who reported that grape supplementation helped to restore the rhythmic expression of core clock genes, including ARNTL. Supporting evidence indicates that ARNTL influences innate immune and inflammatory gene expression, including responses to TLR4 activation in macrophages and modulation of inflammatory enhancers, which ties the circadian clock to NF-κB–mediated signaling [52,53].

In this study, Cisp elevated COX-2 gene expression compared to the control group. COX2 is increased in infertile conditions and testicular pathology; its up-regulation is associated with inflammatory cytokine signaling [54]. Previous studies have shown that inhibiting COX2 expression can alleviate Cisp toxicity [55,56]. Our data demonstrated that *Vitis vinifera* L. extract significantly decreased COX-2 gene expression compared to the Cisplatin group. Previous studies indicated that *Vitis vinifera* extract could attenuate the inflammatory reaction in various cardiovascular diseases, including atherosclerosis [57], diabetic cardiomyopathy [58]. It was also found to relieve inflammation in the pulmonary arterial hypertension model through inhibition of COX-2, as demonstrated by [50]. In this study, Cisp increased GSK-3B gene expression compared to the control group. These results are in accordance with previous data showing that Cisp treatment in both in vivo (animal) and in vitro (tubular epithelial cell) models directly activates GSK-3β, resulting in renal inflammation and nephrotoxicity, whereas targeting GSK-3β by either pharmacological GSK-3β inhibitors or genetic transduction of GSK-3β short-hairpin RNA impeded Cisp-induced cytotoxicity [59].

On the other hand, our data demonstrated that Cisp significantly decreased PI3K gene expression. These results were in agreement with Chang et al. [60] who showed that Cisplatin suppresses the PI3K/AKT/mTOR pathway in testicular cells, indicated by reduced levels of PI3K. Another study by Kuwana et al. [61] showed that mice lacking PI3K showed more severe kidney damage and apoptosis following Cisp treatment, indicating that PI3K/AKT signaling normally acts to protect against Cisp-induced cell death.

It is hypothesized that Cisp may deactivate PI3K/AKT to activate GSK-3β [59]. The PI3K/AKT pathway promotes germ cell survival and Sertoli/Leydig cell function; conversely, reduced PI3K/AKT tone can activate GSK3β, favoring apoptosis and metabolic stress. Our PI3K–GSK3B gene expression pattern, therefore, plausibly intersects with the ROS–R-inflammation milieu to amplify germ cell loss in the Cisplatin context.

Conversely, in this study, when *Vitis vinifera* extract was given alongside Cisp, it significantly increased the expression of PI3K compared to the Cisp group. Chang, Tian, Zhang, Liu, Gao, Li, Liu, Hou, Li and Li [60] reported that *Vitis vinifera* extract reversed the down-regulation of PI3K expression in the testis induced by Cisp. In addition, our results showed that *Vitis vinifera* extract significantly decreased the expression of GSK3B compared to the Cisp group, suggesting that grape seed extract could mitigate apoptosis and inflammation in the testis by inhibiting Cisp-driven GSK3β activation by increasing PI3K.

The current study also showed that Cisp significantly downregulated the gene expression of miRNA 125-b in testis tissue compared to the control group. It was reported by Li et al. [62] that miR-125b-2 knockout in mouse testis is associated with impaired sperm quality, altered mitochondrial DNA copy number, and changes in genes linked to sperm maturation, indicating miR-125b contributes to spermatogenesis and sperm function. On the other hand, in this study, when *Vitis vinifera* extract was given alongside Cisp, it significantly increased the expression of miRNA 125-b relative to Cisp group. Our findings were consistent with those obtained by Li et al. [63], who found that miR-125b mimics reduce ischemia–reperfusion injury and limit apoptosis by modulating p53 network members in non-testis models such as the brain. In addition, Wang et al. [64] reported that miR-125 protects the myocardium from ischemia/reperfusion injury by preventing p53-mediated apoptotic signaling and suppressing TRAF6-mediated NF-κB activation. This suggests miR-125b can be protective against stress-induced germ cell apoptosis. Furthermore, overexpression of miR-125b has been shown to alleviate lung damage by up-regulating ferroptosis inhibitory protein and down-regulating ferroptosis-promoting protein [64]. Li et al. [65] found that miR-125b directly targets HO-1, and its overexpression suppressed silica nanoparticle-induced ferroptosis in cardiomyocytes, evidenced by lower ROS, Fe^2+^ accumulation, and ACSL4 expression. Additionally, miR-125b-5p has been shown to interfere with TLR4/NF-κB signaling by targeting TNFSF4, leading to reduced NF-κB activation and inflammatory responses in vascular endothelial cells [66]. Other studies report miR-125b targeting NF-κB pathway intermediates (e.g., TRAF6/A20), underscoring its potential role in limiting inflammatory signaling under certain conditions [67].

Treatment with Cisp significantly alters the expression of PCNA, a well-established marker of cell proliferation. In the testes, Cisp administration markedly reduces PCNA expression, a finding closely associated with testicular dysfunction. This impairment is evidenced by increased oxidative stress, inflammatory responses, and apoptotic markers within testicular tissue, in parallel with reduced serum levels of testosterone, LH, and FSH [68,69]. Interestingly, our findings demonstrated a contrasting response in the epididymal epithelium, where PCNA expression was significantly up-regulated following Cisp treatment. PCNA is predominantly expressed during the late G1/S phase of the cell cycle and plays a critical role in DNA replication and repair processes. In response to DNA damage, the activation of p53 signaling can transiently induce PCNA expression to facilitate DNA repair independently of cell cycle progression [70,71]. Accordingly, Cisp-induced DNA damage may activate complex DNA damage response pathways, signaling cascades, and epigenetic regulatory mechanisms that collectively drive PCNA overexpression in the epididymis. Moreover, post-translational modifications of PCNA appear to serve as pivotal regulators that balance apoptotic signaling and DNA repair mechanisms in response to Cisp-mediated toxicity [72,73,74].

### Limitations

Although our findings suggest that biomarker modulation attenuates ferroptosis, future studies employing direct ferroptosis markers—such as lipid ROS staining or transmission electron microscopy to assess mitochondrial morphology—are warranted to confirm the mechanistic role of ferroptosis in this context.

Although the present investigation indicated significant treatment-related variations in the mRNA expression of important regulatory genes, including ACSL4, PI3K, and COX-2, these findings reflect transcriptional changes and were not validated by direct protein-level validation. Accordingly, more research integrating Western blotting or immunohistochemistry analyses are necessary to confirm whether these gene expression changes translate into comparable abnormalities at the protein level and functional activity.

## 4. Materials and Methods

### 4.1. The Chemicals

Cis (Cis-diaminedichloroplatinum II) was bought from Macklin Chemicals Company (Shanghai, China). The Biodiagnostics Company in Dokki, Giza, Egypt, provided the kits used for the biochemical analysis. High analytical grade reagents were also used.

### 4.2. Plant Materials

Fruits of *Vitis vinifera* L. (Red grape) were acquired from a local market in Tanta, Al-Gharbia governorate, Egypt. A staff member from Tanta University’s Botany Department, Faculty of Science, identified it.

### 4.3. Preparation of Crude Vitis vinifera L. Seeds Extract

*Vitis vinifera* seeds were manually separated, rinsed with distilled water, and air-dried. A voucher specimen (PG-9-3-HD3) was deposited in the Pharmacognosy Department herbarium at Tanta University, Tanta. The dried seeds (100 g) were finely ground and subjected to triple extraction with an acetone–water mixture (7:3, *v*/*v*) until exhaustion by maceration at ambient temperature (≈25 °C) under continuous shaking, followed by filtration through Whatman filter paper. The pooled extracts were concentrated by vacuum evaporation to remove acetone, and the remaining aqueous fraction was freeze-dried to yield the dried crude extract (15 gm, 3% *w*/*w*) [75,76].

### 4.4. QTOF-HRMS/MS

The produced *Vitis vinifera* seeds extract was subjected to a negative mode analysis using Quadrupole Time-of-Flight High-Resolution Mass Spectrometry (QTOF-HRMS/MS) as previously described [77]. Compound annotation was performed following the identification confidence levels proposed by Schymanski et al. [78]. Authentic standards were not analyzed in the present study; therefore, no Level 1 (confirmed structure) identifications were assigned. Metabolites whose MS/MS spectra showed high-quality matches with public spectral libraries (METLIN, MassBank, GNPS, and mzCloud), including consistent precursor ions, neutral loss patterns, and characteristic fragment ions, were assigned as Level 2 (putatively identified compounds). Compounds exhibiting MS/MS fragmentation patterns supporting the general chemical structure (e.g., flavonoid aglycones, glycosidic sugars, phenolic acids, proanthocyanidin units), but lacking sufficient evidence to distinguish between positional/isomeric variants, were classified as Level 3 (tentative candidates). No Level 4 annotations were included, as MS/MS data were successfully acquired for all detected features. The confidence levels associated with each metabolite are presented in Table 1.

### 4.5. Experimental Animals

Prior to the experiment, forty male albino Wistar rats weighing between 150 and 180 g were kept under the specified conditions (12 h light/dark cycle, temperature 22 ± 3 °C, and 50% humidity) for two weeks to adapt. They were given water and a regular meal. The experimental techniques were authorized by the Institutional Animal Care and Use Committee (IACUC) of Alexandria University in Egypt under approval number ALEXU-IACUC, 13-2025-07-12/359. Every procedure adhered to the ARRIVE standards [79]. Careful handling to avoid unnecessary disturbances, compression, pressure, or painful manipulation was among the tight steps implemented to reduce the number of animals used and their suffering.

### 4.6. Experimental Design

Forty adult male albino rats were divided into four groups, with ten rats per group. Group 1 (Control): Rats received an oral administration of 1 mL of saline for 18 days. Group 2 (*Vitis vinifera* extract): Rats received a daily oral dose of *Vitis vinifera* L. extract (100 mg/kg) for 18 days [80]. Group 3 (Cisp): Rats received an intraperitoneal injection of Cisp (7 mg/kg) as a single dose on day 15. The Cisp dose was selected based on a previous study [69,81]. Groups 4 (*Vitis vinifera* L. extract + Cisp): Rats received *Vitis vinifera* L. extract (100 mg/kg) for 18 consecutive days and on day 15, they were concomitantly injected with Cisp (7 mg/kg; i.p, single dose).

### 4.7. Blood and Tissue Collection

Following euthanasia with isoflurane, blood samples were collected from the aorta into plain tubes and allowed to clot for 30 min at a slanted position. The samples were then centrifuged for 15 min, and the separated serum was transferred into clean Eppendorf tubes for subsequent biochemical studies. Testicles from all animals were carefully excised: one was fixed in 10% neutral buffered formalin for histopathological and immunohistochemical examinations, while the other was rinsed in saline, homogenized, and stored at −80 °C for later biochemical and molecular investigations.

### 4.8. Sperm Parameters Measurements

Sperm samples were taken from the caudal area of the epididymis (about 1 cm from the distal end) and minced in 2 mL of phosphate-buffered saline (PBS). To enhance sperm dispersion, the suspension was incubated at 37 °C for 30 min. After incubation, the solution was gently stirred to achieve homogeneity. A light microscope was used at 400× magnification to evaluate sperm count and motility.

### 4.9. Sperm Counting

To gather epididymal sperm, the cauda epididymis was sliced with a sharp scalpel and flushed out in a sterile watch glass. In a watch glass, the released epididymal sperm were gathered. A hemocytometer and a pipette used for RBC counts were used to count epididymal sperm. After removing a drop of diluted cauda epididymal content up to the 0.5 mark, the pipette was filled to the 101 with 4% formalin (to destroy the sperms) and 5% sodium bicarbonate solution (to break up the mucus droplets in the diluting pipette). By holding the pipette’s ends between the thumb and index fingers and shaking it vigorously, the contents were combined. A tiny quantity of the diluted sperm solution was then put near the edge of the hemocytometer’s cover slide after a few fluid droplets were blown out. Bearden and Fuquay [82] method was used to estimate the sperm cell count in milliliters.

### 4.10. Sperm Motility

On a heated microscope stage, a dry, clean slide was set up. Vas deferens was squeezed using a sharp scalpel. One drop of physiological saline was combined with one drop of recently undiluted content of vas-difference on the heated slide. Low-power objective lens of the light microscope (X10) was utilized to assess the percentage of progressively motile sperm using Bearden and Fuquay [82] approach.

### 4.11. Serum FSH, LH, and Testosterone Measurement

Quantitative evaluations of follicular-stimulating hormone (FSH) and luteinizing hormone (LH) were made in serum using special ELISA kits from CUSABIO Co. Ltd. (Wuhan, China) (Cat. No CSB-E069r and CSB-E12654r, respectively). Following the manufacturer’s guidelines, an ELISA microplate reader was used at 450 nm. Also, testosterone level was analyzed quantitatively in serum using specific ELISA kits (Cat. No CSB-E05100r; CUSABIO Co.) according to Tietz [83].

### 4.12. Testicular Oxidative and Antioxidant Biomarkers Evaluation

Testicular malondialdehyde (MDA) was measured using Draper and Hadley [84] technique. Estimation of testicular superoxide dismutase (SOD), and catalase (CAT) activities were assessed according to the techniques outlined by Nishikimi et al. [85] and Beers and Sizer [86], respectively, while total (tGSH), reduced (GSH), and oxidized glutathione (GSSG) contents were estimated based on the methodology of Griffith [87]. All prior parameters have been evaluated spectrophotometrically using commercial kits from Bio-diagnostic Co. (Dokki, Giza, Egypt), as directed by the manufacturer.

The NRF2 was quantitatively measured in the testicular homogenate using a rat Nrf2 ELISA kit BioSource Co. (San Diego, CA, USA) Cat. No. MBS752046. Also, the hemoxgenase enzyme-1(Ho-1) was quantitatively measured in the testicular homogenate using the kit CUSABIO Co. Cat. No. CSB.E08267r. Estimation of glutathione peroxidase 4 (GPx4) activities was assessed according to the techniques outlined by Flohé and Günzler [88], respectively. The rat 8_hydroxy_desoxyguanosine (8-OHdG) was quantitatively measured in the testicular homogenate using 8-OHdG ELISA KIT CUSABIO Co., Cat. No. (CSB_E10526r). All trials were prepared according to the manufacturer’s directions.

### 4.13. Testicular Pro-Inflammatory Cytokines and Some Ferroptosis Biomarkers Assessments

TNF-α was quantified in testicular homogenate using a rat TNF-α ELISA kit from Chongqing Biospes Co., Ltd. (Chongqing, China) (Cat. No. BEK1214), while interleukin-1ẞ (IL-1β) was measured quantitatively using a rat IL-1β ELISA kit from CUSABIO Co. (Cat. No CSB-E08055r). Furthermore, a rat NF-κB_p65 ELISA kit from Chongqing Biospes Co., Ltd. was used to quantitatively identify the nuclear factor kappa light chain enhancer of activated B cells (NF-κB) in testicular homogenates (Cat. No. BYEK3040). All trials were prepared in accordance with the manufacturer’s directions.

The ferritin was quantitatively evaluated in the testicular homogenate using Rat Ferritin ELISA kit AbCam Co. (Waltham, MA, USA) Cat. No. ab157732. Also, the cathepsin was quantitatively measured in the testicular homogenate using the Ctsb ELISA kit, Ec3.4.22.1 Cat. No. CSB_EL006185RA, CUSABIO Co. All trials were prepared in accordance with the manufacturer’s directions.

### 4.14. Gene Expression Analysis Using Quantitative Real-Time Polymerase Chain Reaction (qPCR)

Total RNA was extracted from the testis using the RNeasy Mini kit (QIAGEN, Hilden, Germany) according to the manufacturer’s protocol for all measured genes. The expression of miR-125b in the testis tissue was assayed using TaqMan^®^ miR-125b (Thermo Fisher Scientific, Waltham, MA, USA, Cat.no. 4427975, ID: 000449) kit. Then, 5 μg total RNA was converted to cDNA by TOPscript™ RT DryMIX (dT18/dN6 plus) kit according to the manufacturer’s instructions (Enzynomics Co Ltd., Daejeon, Republic of Korea, cat number RT220). One μL of the cDNA was used for quantitative PCR using ViPrime PLUS Taq qPCR Green Master Mix I (Vivantis Technologies, Subang Jaya, Malaysia, cat. Number QLMM12), as described by the manufacturer. The sequence of primers of target genes (Biosearch Technologies Co., Petaluma, CA, USA) used in qPCR are shown in Table 2. PCR instrument was adjusted according to the program: 95 °C, 1 min, followed by 40 cycles (95 °C for 5 sec for denaturation and 55 °C for 10 sec (annealing/extension). Data was collected using Bio-Rad CFX Maestro version 2.3 (Bio-Rad, Inc., Hercules, CA, USA). Each sample was quantified relative to the expression of the reference gene (18S rRNA) for all measured genes, while U6 (cat no. 4427975, ID: 001973) was utilized as a reference gene for measuring miR-125b. C_t_ (threshold cycle) values of the sample were calculated, and transcript levels were analyzed by 2^−ΔΔCt^ method [89].

### 4.15. Relative Quantification of the Expression of miR-155 Using PCR

MiR-125b expression in the testicular tissue was assayed using TaqMan^®^ miR-125b (Thermo Fisher Scientific, Cat. no. 4427975, ID: 000449) kit and using U6 as a reference gene (cat no. 4427975, ID: 001973). Quantitative PCR began with an initial denaturation at 95 °C for 10 min and amplification via 45 cycles of PCR as follows: Denaturation at 95 °C for 5 s, annealing at 55 °C for 15 s, and then extension at 60 °C for 15 s. Amplification, data acquisition, and analysis were performed on CFX96 Touch Deep Well Real-Time PCR Detection System (Bio-Rad Laboratories, CA, USA). The values of threshold cycle (Ct) were determined by CFX Maestro™ Software version 1.1 (Bio-Rad laboratories, CA, USA). The relative change in miR-21 in samples was determined using the 2^−ΔΔCt^ method and normalized to the reference U6 as described previously.

### 4.16. Histopathologic Examination

For fixed testes and epididymis, the conventional paraffin embedding technique was employed after fixation in Bouin’s solution. The sections were cut into 4 µm thick slices and stained using the Hematoxylin and Eosin (H&E) technique [90]. Determining the extent of testicular injury and the effectiveness of spermatogenesis were evaluated using Cosentino [91] and Johnsen’s grading scores, respectively [92]. Using Cosentino’s grading system, the testis is divided into four groups, ranging from normal testes to necrotic tissue. In Johnsen’s score, a score of 10 denotes normal spermatogenesis, a score of 9 denotes several spermatozoa dispersed throughout the germinal epithelium, and a score of 8 denotes a low number of spermatozoa. Conversely, a score of 1 indicates that there is no cellular structure at all within the seminiferous tubules, and a score of 7 to 2 indicates that maturation is complete. Epididymal lesions were evaluated semiquantitatively using Gibson-Corley et al. [93]. Ten randomly chosen pictures of defects (vacuolation and interstitial fibrosis) were taken from each slide for each rat, and the mean was then calculated. The following scoring system was used to evaluate the scored parameters: 0 = normal, 1 ≤ 25%, 2 = 26% to 50%, 3 = 51% to 75%, and 4 = 76% to 100%.

### 4.17. Immunohistochemical Protein Assay

The slides were deparaffinized, rehydrated, and submerged in a 10 mM sodium citrate buffer solution before being heated in a microwave to aid antigen retrieval. The endogenous peroxidase enzyme was inactivated by incubating it with 3% hydrogen peroxide in absolute methanol for 30 min at 4 °C and then washing with PBS. Nonspecific binding was avoided by using 10% normal blocking serum for 60 min at room temperature. After being exposed to nuclear factor kappa beta (NF-kB p65, ab16502, 1:200, Abkam, Cambridge, UK) and proliferating cell nuclear antigen (PCNA, Catalog # (F-2): sc-25280, 1:50 dilution, Santa Cruz, CA, USA) for an entire night at 4 °C, the first antibodies were rinsed with PBS and incubated with a second antibody for 60 min. Following PBS cleaning, the slices were treated with the streptavidin–peroxidase conjugate for half an hour. The streptavidin–biotin complex was then incubated in a 3, 3′-diaminobenzidine tetrahydrochloride-H2O2 solution for three minutes at 7.0 pH. Following a thorough cleaning with distilled water, the slices were colored using Mayer’s hematoxylin [94]. Ten randomly selected locations from five animals in each group were used to quantify the area percentage of NF-kB and caspase-3 immune-expression using FIJI ImageJ software version 1.54f (National Institutes of Health, Bethesda, MD, USA).

### 4.18. Statistical Analysis

Data normality was assessed using the Shapiro–Wilk test, while the homogeneity of variances was examined using Levene’s test. Values were expressed as mean ± SE, and the Tukey–Kramer post hoc test was used after analysis of variance (ANOVA) for group comparisons. After the Kruskal–Wallis test, Dunn’s test was used to examine non-parametric lesions. A significance level of *p* < 0.05 was used. (GraphPad^®^ program Inc., Version 10.3.1, San Diego, CA, USA) The Prism program was used for all statistical analyses [95].

## 5. Conclusions

The present study provides compelling evidence that *Vitis vinifera* extract exerts a robust protective effect against Cisp-induced testicular toxicity in rats. Cisp administration markedly impaired reproductive performance, as reflected by deterioration in sperm characteristics, suppression of reproductive hormones, induction of oxidative stress, activation of ferroptosis, and stimulation of inflammatory cascades, in addition to significant alterations in gene expression profiles associated with cellular stress responses. Co-treatment with *Vitis vinifera* L. extract effectively ameliorated these detrimental changes, restoring sperm quality, hormonal balance, and antioxidant capacity while attenuating inflammation and ferroptosis. Moreover, *Vitis vinifera* L. extract normalized the expression of critical regulatory genes, underscoring its multifaceted mechanism of action. As *Vitis vinifera* L. extract is a relatively nontoxic natural product, it could be useful as a prophylactic measure or adjuvant therapy in controlling Cisp-induced testicular damage. Although this experimental study provides valuable mechanistic insights, further investigations are warranted to validate these effects in clinical settings, optimize dosing strategies, and explore potential synergistic interactions with standard treatments. Collectively, our results open avenues for the development of *Vitis vinifera* L. extract-based interventions aimed at mitigating chemotherapy-induced gonad toxicity and improving the quality of life of cancer patients.

## Figures and Tables

**Table 1:** Natural compounds detected in *Vitis vinifera* seeds extract, using negative mode ionization QTOF-HRMS/MS analysis *.

No.	RT (min)	Precursor, *m*/*z* [M-H]^−^	Error (ppm)	Tentative Assignment (Compound Name)	Molecular Formula	Ontology	Confidence Level	Basis for Assignment
1	0.57	389.1260	3.2	Resveratroloside (Resveratrol glycoside)	C_20_H_22_O_8_	Stilbenoid glucoside	Level 2	Resveratrol fragments + sugar neutral loss
2	0.59	451.1235	−2.4	Catechin 5-O-glucoside	C_21_H_24_O_11_	Flavanol glucoside	Level 3	Aglycone confirmed; glycosylation position uncertain
3	0.72	289.0706	−4.0	Catechin (Cianidanol)	C_15_H_14_O_6_	Flavanol	Level 2	Strong library MS/MS match
4	1.24	355.1042	2.0	Ferulic acid β-glucoside	C_16_H_20_O_9_	Phenolic glycosides	Level 2	Ferulic acid diagnostic ions + sugar loss
5	1.62	353.0884	1.6	Chlorogensaure (Neochlorogenic acid)	C_16_H_18_O_9_	Phenolic acids	Level 2	Characteristic caffeoylquinic acid fragmentation
6	1.84	771.1996	0.8	Quercetin 3-O-gentiobioside-7-O-rhamnoside	C_33_H_40_O_21_	Flavonol glycosides	Level 3	Multiglycosylation prevents structural confirmation
7	2.15	193.0515	4.5	Ferulic acid	C_10_H_10_O_4_	Ferulic acids	Level 2	Excellent match with libraries
8	3.23	579.1363	1.3	Kaempferol 3-O-α-arabinopyranosyl (1‴-6″)-β- glucopyranoside	C_26_H_28_O_15_	Flavonol O-glycosides	Level 3	Linkage and sugar identity tentative
9	3.58	609.1465	0.7	kaempferol 3-O-sophoroside	C_27_H_30_O_16_	Flavonol O-glycosides	Level 2/3	Aglycone clear; disaccharide linkage tentative
10	3.68	801.2086	−1.1	Isorhamnetin 3-gentiobioside-7-glucoside	C_34_H_42_O_22_	Flavonol Glycosides	Level 3	Multiple sugars; tentative configuration
11	3.69	755.2033	−1.0	Isorhamnetin 3-xylosyl-(1-2)-glucoside-7-rhamnoside	C_33_H_40_O_20_	Methoxyflavonol Glycosides	Level 3	Complex glycoside; tentative
12	3.72	563.1416	1.7	Kaempferol 3-O- α-L-rhamnosyl (1-2)β-D-arabinoside	C_26_H_28_O_14_	Flavonol Glycosides	Level 3	Class-level identification
13	3.93	447.0942	2.0	Luteolin 4′-β-D-O-glucoside	C_21_H_20_O_11_	Flavone Glycoside	Level 2/3	Luteolin fragments clear; position partially tentative
14	4.04	593.1520	1.3	Kaempferol 3-O-glucosyl-(1-2)-rhamnoside	C_27_H_30_O_15_	Flavonol Glycoside	Level 3	Sugar linkage unresolved
15	4.07	197.0465	4.9	Syringic acid	C_9_H_10_O_5_	Gallic acid derivative	Level 2	Simple phenolic; strong match
16	4.08	739.2086	−0.6	Kaempferol 3-neohesperidoside-7-rhamnoside	C_33_H_40_O_19_	Flavonol O-Glycosides	Level 3	Multiglycosylated flavonol
17	4.11	447.0940	1.5	Luteolin 3′-glucoside (Dracocephaloside)	C_21_H_20_O_11_	Flavone O-Glycosides	Level 2	Known MS/MS pattern
18	4.16	725.1940	0.8	Kaempferol 3-apioside-7-rhamnosyl-(1-6)-galactoside	C_32_H_38_O_19_	Flavonol O-Glycosides	Level 3	Multiple sugars; class-level identification
19	4.44	577.1571	1.4	kaempferol 3,7-di-O-α-L-rhamnoside	C_27_H_30_O_14_	Flavone O-Glycosides	Level 3	Di-glycosylation ambiguous
20	4.46	1033.2018	2.4	Galloylated prodelphinidin (trimers)	C_52_H_42_O_23_	Proanthocyanidins	Level 3	Proanthocyanidin class identifiable; composition tentative
21	4.47	533.1310	1.7	Apigenin 6-C-alpha-L-arabinopyranosyl-8-C-beta-D-xylopyranoside	C_25_H_26_O_13_	Flavone C-Glycosides	Level 3	C-glycoside isomerism unresolved
22	4.54	431.0996	2.9	Kaempferol-7-O-alpha-L-rhamnoside	C_21_H_20_O_10_	Flavone O-Glycosides	Level 2/3	Good match; position not fully confirmed
23	4.64	917.2364	0.8	Kaempferol 7-O-(6-trans-caffeoyl)-beta-glucopyranosyl-(1-3)-alpha-rhamnopyranoside-3-O-beta-glucopyranoside	C_42_H_46_O_23_	Flavone O-Glycosides	Level 3	Highly complex; class-level
24	4.71	593.1529	2.9	Apigenin-7-O-gentiobioside	C_27_H_30_O_15_	Flavone O-Glycosides	Level 2/3	Aglycone clear; sugar linkage tentative
25	4.82	901.2410	0.2	Kaempferol 7-O-(6-trans-p-coumaroyl)-β-glucopyranosyl-(1-3)-α-rhamnopyranoside-3-O-β-glucopyranoside	C_42_H_46_O_22_	Flavone O-Glycosides	Level 3	Class-level only
26	5.20	475.1246	0.0	Luteolin 7,3′-dimethyl ether 5-glucoside	C_23_H_24_O_11_	Flavone O-Glycosides	Level 2/3	Known fragmentation; methylation pattern tentative
27	6.03	431.0988	1.1	kaempferol-3-O-α-L-rhamnoside	C_21_H_20_O_10_	Flavone O-Glycosides	Level 2/3	Common glycoside; minor ambiguity
28	6.77	301.0344	−3.1	Quercetin Dihydrate	C_15_H_10_O_7_	Flavonols	Level 2	Aglycone match; hydration commonly observed
29	7.28	329.0662	−1.4	Quercetin 3,4′-dimethyl ether	C_17_H_14_O_7_	Flavonols	Level 2/3	Fragmentation consistent with dimethyl quercetin
30	7.67	461.1093	0.7	Isorhamnetin 3-O-Rhamnoside	C_22_H_22_O_11_	Flavonol O-Glycosides	Level 2	Good MS/MS match
31	7.68	489.1048	2.0	Kaempferol-3-O-(6′′-O-acetyl)-β-D-glucopyranoside	C_23_H_22_O_12_	Flavone O-Glycosides	Level 3	Acetylation position uncertain
32	11.54	499.1236	−1.0	Catechin Pentaacetate	C_25_H_24_O_11_	Flavanols	Level 2/3	Aglycone identifiable; acetylation tentative
33	14.53	479.0808	−1.8	Myricetin 3-O-α-D-glucopyranoside	C_21_H_20_O_13_	Flavonol O-Glycosides	Level 2	Strong literature/library match
34	15.22	311.1709	1.3	(2S,3R)-trans-Caftaric acid	C_13_H_12_O_9_	Esterified phenolic acid	Level 2	Known tartaric ester fragmentation
35	15.49	479.0853	4.6	Quercetin 6-glucoside	C_21_H_20_O_13_	Flavonol C-Glycosides	Level 2/3	Aglycone clear; position tentative
36	16.00	325.1859	2.3	Trans-p-Coumaric acid 4-glucoside	C_15_H_18_O_8_	Phenolic glycosides	Level 2	Clear diagnostic ions
37	17.76	763.2097	0.8	Kaempferol-3-O-lysimachiatrioside	C_35_H_40_O_19_	Flavone O-Glycosides	Level 3	Rare trisaccharide; tentative
38	18.73	504.1272	3.3	Peonidin 3-O-(6-O-acetyl-β-D-glucoside)	C_24_H_25_O_12_^+^	Anthocyanin cation	Level 2/3	Anthocyanin fragmentation clear; acetylation uncertain
39	19.44	563.1412	1.1	Kaempferol 3-O-α-L-rhamnosyl (1-2)β-D-xyloside	C_26_H_28_O_14_	Flavone O-Glycosides	Level 3	Glycosylation linkage unresolved
40	21.65	549.0878	−1.4	Quercetin 3-O-(6″-o-malonyl)-β-D-glucoside	C_24_H_22_O_15_	Flavonol O-Glycosides	Level 3	Malonylation position tentative
41	23.87	455.3535	−0.4	Oleanolic Acid	C_30_H_48_O_3_	Pentacyclic triterpenoid	Level 2	Diagnostic triterpenoid MS/MS pattern

RT: retention time; *m*/*z*: mass-to-charge ratio; ppm: 10^−6^, * Compound identifications were assigned according to the Schymanski confidence level system. Level 2 annotations correspond to compounds with MS/MS spectra that exhibit strong matches to public libraries or literature data. Level 3 annotations represent metabolites for which MS/MS data support the overall chemical class (e.g., flavonol glycosides, proanthocyanidins), but do not permit unambiguous assignment of positional or isomeric features, particularly in the case of highly glycosylated or acylated flavonoids. No Level 1 (reference standard-confirmed) identifications were obtained.

**Table 2:** Primer sets of studied genes.

Gene	Accession No.	Primer Sequence	
**PI3K**	NM_013005.2	**F**	TGCTATGCCTGCTCTGTAGTGGT
		**R**	GTGTGACATTGAGGGAGTCGTTG
**GSK3B**	NM_032080.1	**F**	GGAACTCCAACAAGGGAGCA
		**R**	TTCGGGGTCGGAAGACCTTA
**ARNTL**	NM_024362.2	**F**	ACCTCGCAGAATGTCACAGGCA
		**R**	CTGAACCATCGACTTCGTAGCG
**ACSL4**	NM_001431651.1	**F**	CCTTTGGCTCATGTGCTGGAAC
		**R**	GCCATAAGTGTGGGTTTCAGTAC
**COX2**	NM_017232.4	**F**	GCGACATACTCAAGCAGGAGCA
		**R**	AGTGGTAACCGCTCAGGTGTTG
**miR-125b**	MIMAT0000830	UCCCUGAGACCCUAACUUGUGA	
**18s rRNA**	NR_046237.2	**F**	GTAACCCGTTGAACCCCATT

## Data Availability

The original contributions presented in this study are included in the article. Further inquiries can be directed to the corresponding author.
